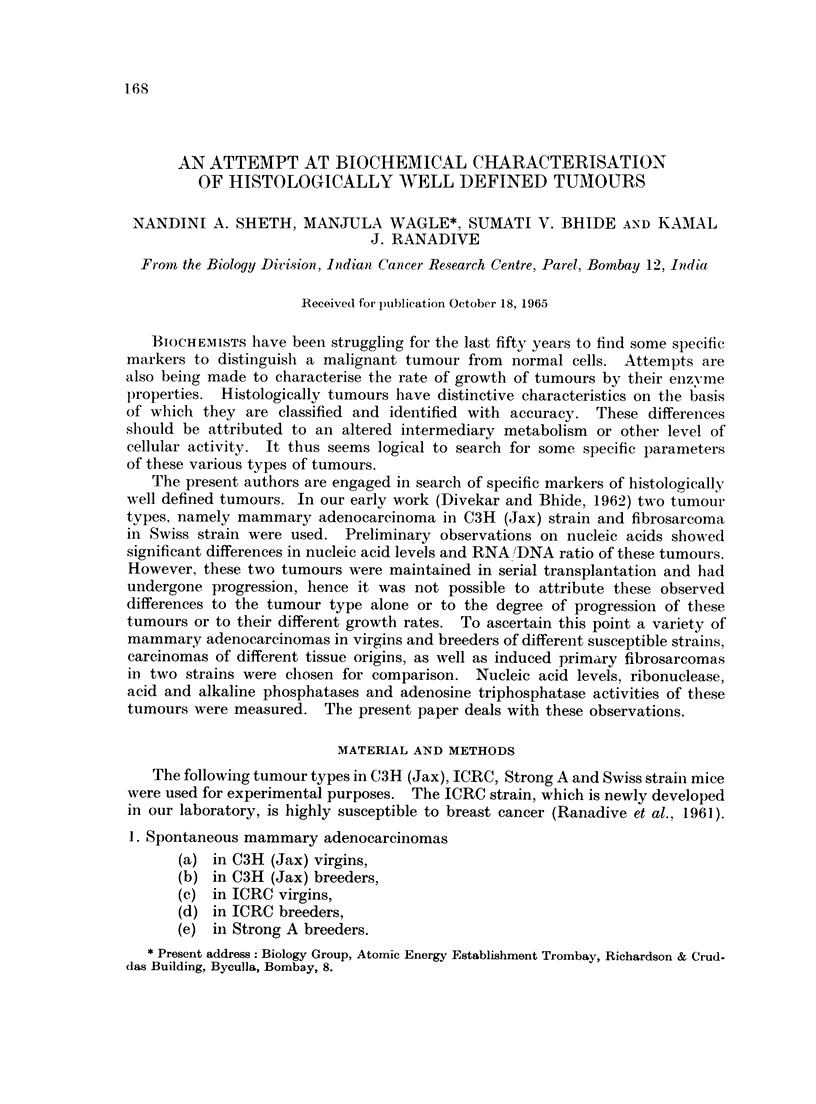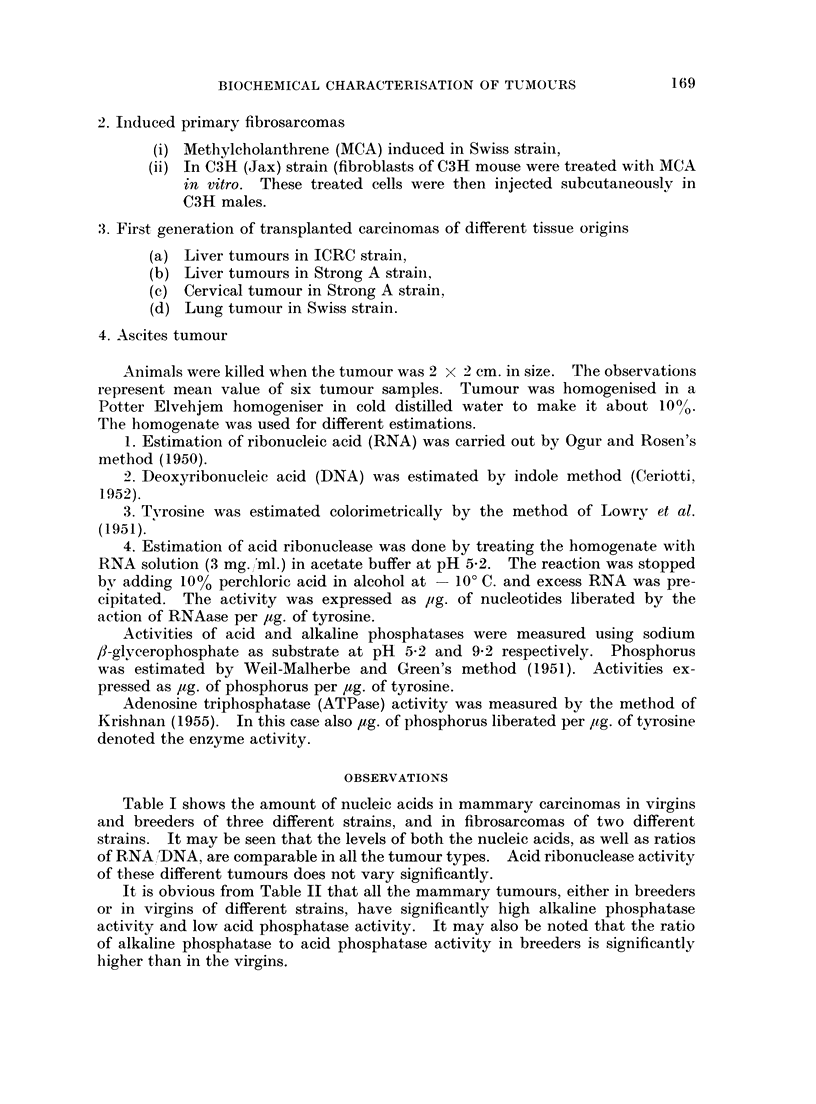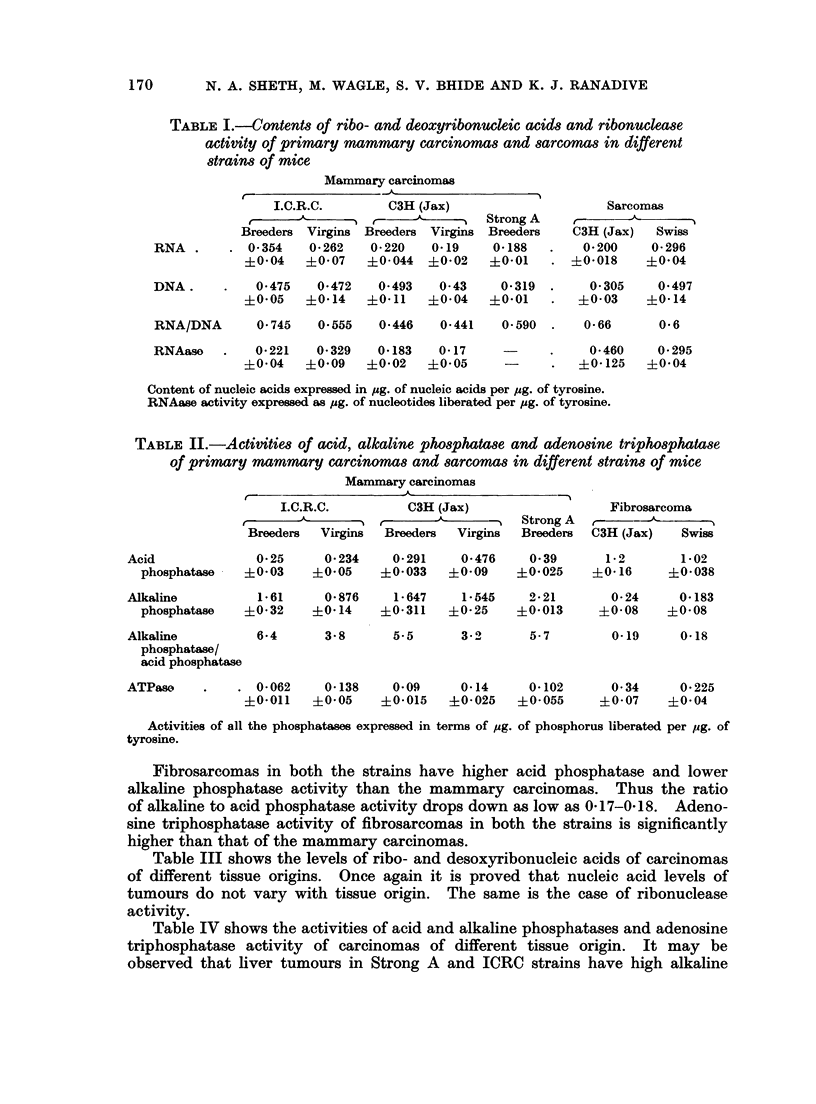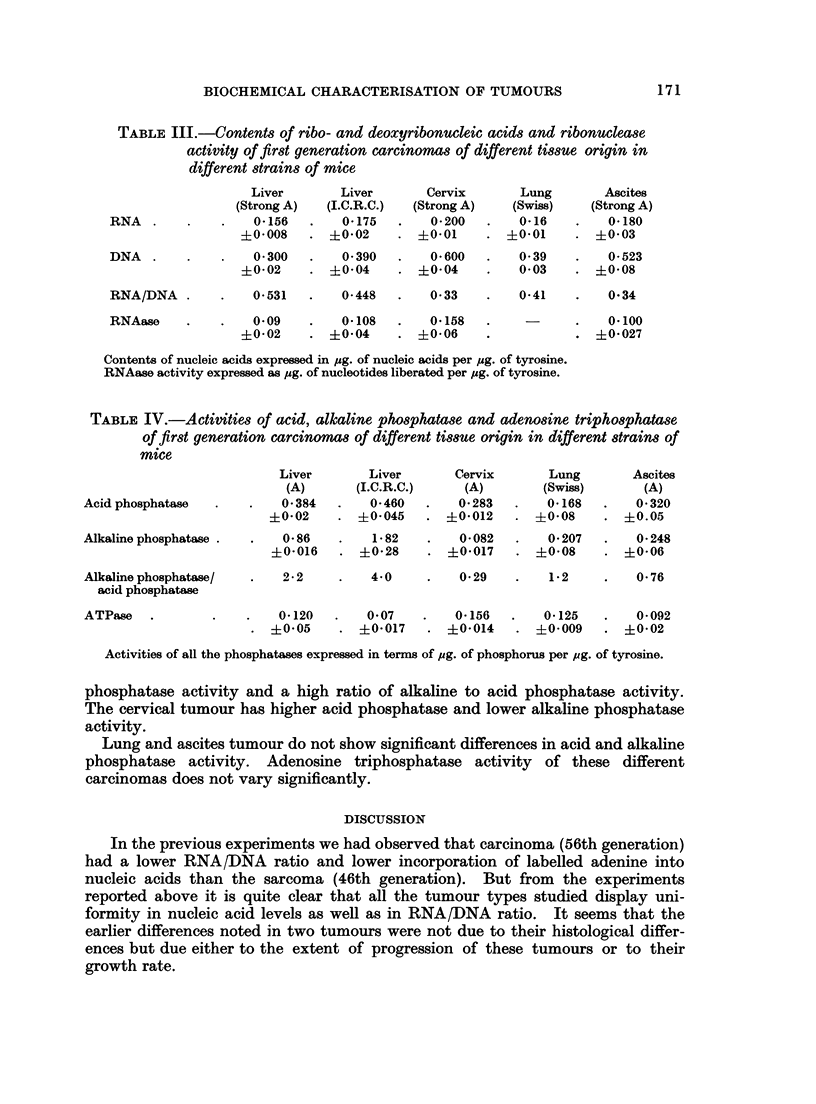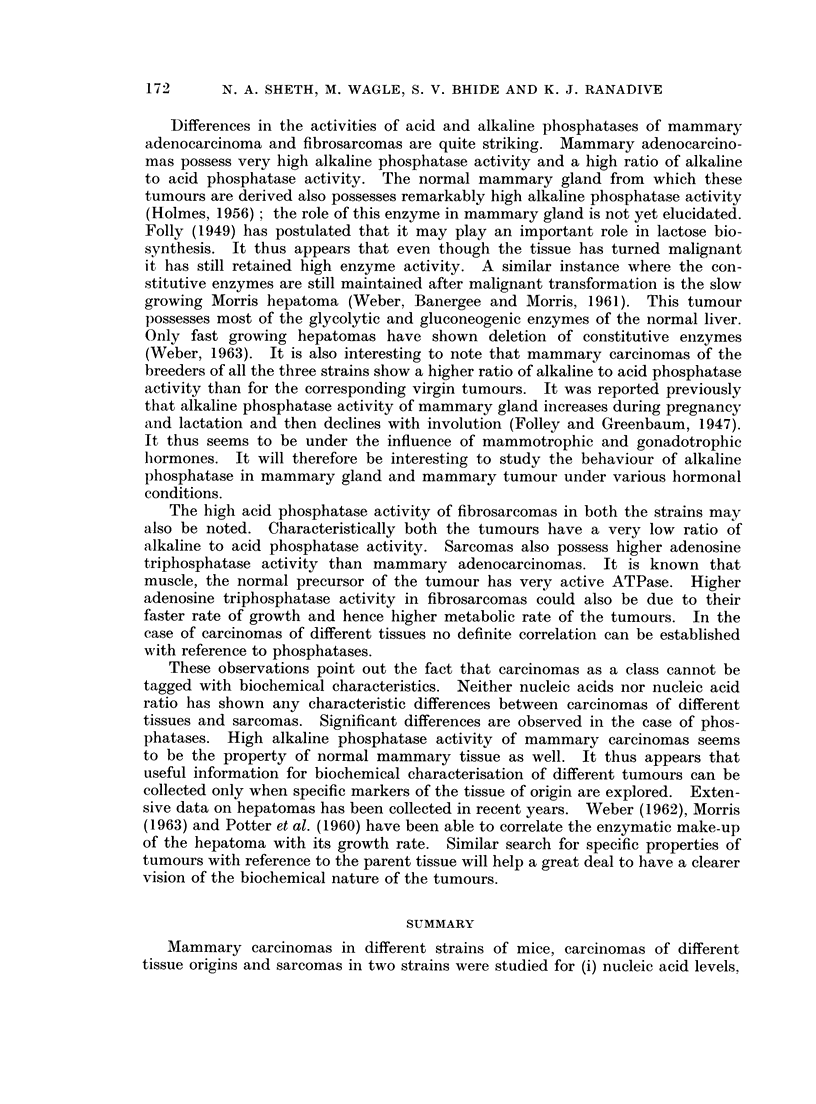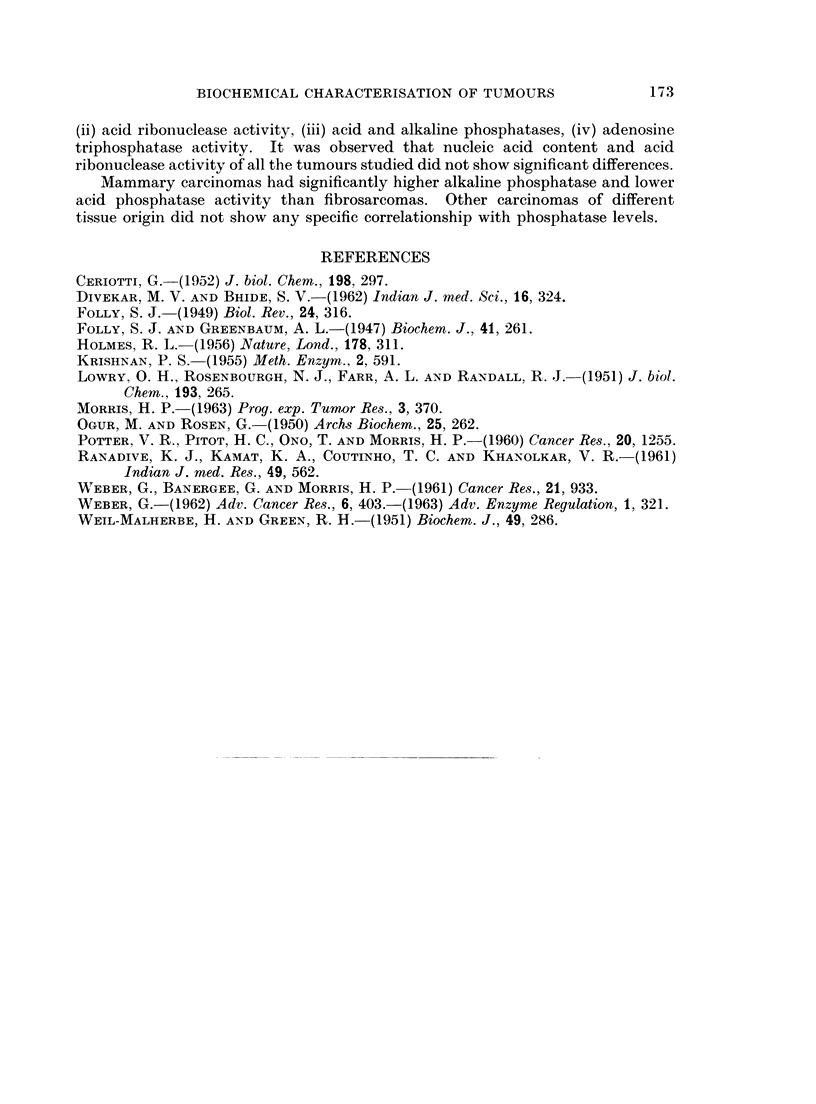# An attempt at biochemical characterisation of histologically well defined tumours.

**DOI:** 10.1038/bjc.1966.19

**Published:** 1966-03

**Authors:** N. A. Sheth, M. Wagle, S. V. Bhide, K. J. Ranadive


### AN ATTEMPT AT BIOCHEMICAL CHARACTERISATION OF HISTOLOGICALLY WELL DEFINED TUMOURS

NANDINI A. SHETH, MANJULA WAGLE*, SUMATI V. BHIDE AND KAMAL J. RANADIVE

*From the Biology Division, Indian Cancer Research Centre, Parel, Bombay* 12, *India*

Received for publication October 18, 1965

BIOCHEMISTS have been struggling for the last fifty years to find some specific markers to distinguish a malignant tumour from normal cells. Attempts are also being made to characterise the rate of growth of tumours by their enzyme properties. Histologically tumours have distinctive characteristics on the basis of which they are classified and identified with accuracy. These differences should be attributed to an altered intermediary metabolism or other level of cellular activity. It thus seems logical to search for some specific parameters of these various types of tumours.

The present authors are engaged in search of specific markers of histologically well defined tumours. In our early work (Divekar and Bhide, 1962) two tumour types, namely mammary adenocarcinoma in C3H (Jax) strain and fibrosarcoma in Swiss strain were used. Preliminary observations on nucleic acids showed significant differences in nucleic acid levels and RNA/DNA ratio of these tumours. However, these two tumours were maintained in serial transplantation and had undergone progression, hence it was not possible to attribute these observed differences to the tumour type alone or to the degree of progression of these tumours or to their different growth rates. To ascertain this point a variety of mammary adenocarcinomas in virgins and breeders of different susceptible strains, carcinomas of different tissue origins, as well as induced primary fibrosarcomas in two strains were chosen for comparison. Nucleic acid levels, ribonuclease, acid and alkaline phosphatases and adenosine triphosphatase activities of these tumours were measured. The present paper deals with these observations.

### MATERIAL AND METHODS

The following tumour types in C3H (Jax), ICRC, Strong A and Swiss strain mice were used for experimental purposes. The ICRC strain, which is newly developed in our laboratory, is highly susceptible to breast cancer (Ranadive *et al.*, 1961).

1. Spontaneous mammary adenocarcinomas
    - (a) in C3H (Jax) virgins,
    - (b) in C3H (Jax) breeders,
    - (c) in ICRC virgins,
    - (d) in ICRC breeders,
    - (e) in Strong A breeders.

* Present address : Biology Group, Atomic Energy Establishment Trombay, Richardson & Cruddas Building, Byculla, Bombay, 8.

2. Induced primary fibrosarcomas
   (i) Methylcholanthrene (MCA) induced in Swiss strain,
   (ii) In C3H (Jax) strain (fibroblasts of C3H mouse were treated with MCA *in vitro*. These treated cells were then injected subcutaneously in C3H males.

3. First generation of transplanted carcinomas of different tissue origins
   (a) Liver tumours in ICRC strain,
   (b) Liver tumours in Strong A strain,
   (c) Cervical tumour in Strong A strain,
   (d) Lung tumour in Swiss strain.

4. Ascites tumour

Animals were killed when the tumour was 2 × 2 cm. in size. The observations represent mean value of six tumour samples. Tumour was homogenised in a Potter Elvehjem homogeniser in cold distilled water to make it about 10%. The homogenate was used for different estimations.

1. Estimation of ribonucleic acid (RNA) was carried out by Ogur and Rosen's method (1950).

2. Deoxyribonucleic acid (DNA) was estimated by indole method (Ceriotti, 1952).

3. Tyrosine was estimated colorimetrically by the method of Lowry *et al.* (1951).

4. Estimation of acid ribonuclease was done by treating the homogenate with RNA solution (3 mg./ml.) in acetate buffer at pH 5·2. The reaction was stopped by adding 10% perchloric acid in alcohol at − 10° C. and excess RNA was precipitated. The activity was expressed as μg. of nucleotides liberated by the action of RNAase per μg. of tyrosine.

Activities of acid and alkaline phosphatases were measured using sodium β-glycerophosphate as substrate at pH 5·2 and 9·2 respectively. Phosphorus was estimated by Weil-Malherbe and Green's method (1951). Activities expressed as μg. of phosphorus per μg. of tyrosine.

Adenosine triphosphatase (ATPase) activity was measured by the method of Krishnan (1955). In this case also μg. of phosphorus liberated per μg. of tyrosine denoted the enzyme activity.

### OBSERVATIONS

Table I shows the amount of nucleic acids in mammary carcinomas in virgins and breeders of three different strains, and in fibrosarcomas of two different strains. It may be seen that the levels of both the nucleic acids, as well as ratios of RNA/DNA, are comparable in all the tumour types. Acid ribonuclease activity of these different tumours does not vary significantly.

It is obvious from Table II that all the mammary tumours, either in breeders or in virgins of different strains, have significantly high alkaline phosphatase activity and low acid phosphatase activity. It may also be noted that the ratio of alkaline phosphatase to acid phosphatase activity in breeders is significantly higher than in the virgins.

TABLE I.—*Contents of ribo- and deoxyribonucleic acids and ribonuclease activity of primary mammary carcinomas and sarcomas in different strains of mice*

| | Mammary carcinomas | | | | | Sarcomas | |
|---|---|---|---|---|---|---|---|
| | I.C.R.C. | | C3H (Jax) | | Strong A | | |
| | Breeders | Virgins | Breeders | Virgins | Breeders | C3H (Jax) | Swiss |
| RNA | 0·354 ±0·04 | 0·262 ±0·07 | 0·220 ±0·044 | 0·19 ±0·02 | 0·188 ±0·01 | 0·200 ±0·018 | 0·296 ±0·04 |
| DNA | 0·475 ±0·05 | 0·472 ±0·14 | 0·493 ±0·11 | 0·43 ±0·04 | 0·319 ±0·01 | 0·305 ±0·03 | 0·497 ±0·14 |
| RNA/DNA | 0·745 | 0·555 | 0·446 | 0·441 | 0·590 | 0·66 | 0·6 |
| RNAase | 0·221 ±0·04 | 0·329 ±0·09 | 0·183 ±0·02 | 0·17 ±0·05 | — — | 0·460 ±0·125 | 0·295 ±0·04 |

Content of nucleic acids expressed in μg. of nucleic acids per μg. of tyrosine.

RNAase activity expressed as μg. of nucleotides liberated per μg. of tyrosine.

TABLE II.—*Activities of acid, alkaline phosphatase and adenosine triphosphatase of primary mammary carcinomas and sarcomas in different strains of mice*

| | Mammary carcinomas | | | | | Fibrosarcoma | |
|---|---|---|---|---|---|---|---|
| | I.C.R.C. | | C3H (Jax) | | Strong A | | |
| | Breeders | Virgins | Breeders | Virgins | Breeders | C3H (Jax) | Swiss |
| Acid phosphatase | 0·25 ±0·03 | 0·234 ±0·05 | 0·291 ±0·033 | 0·476 ±0·09 | 0·39 ±0·025 | 1·2 ±0·16 | 1·02 ±0·038 |
| Alkaline phosphatase | 1·61 ±0·32 | 0·876 ±0·14 | 1·647 ±0·311 | 1·545 ±0·25 | 2·21 ±0·013 | 0·24 ±0·08 | 0·183 ±0·08 |
| Alkaline phosphatase/ acid phosphatase | 6·4 | 3·8 | 5·5 | 3·2 | 5·7 | 0·19 | 0·18 |
| ATPase | 0·062 ±0·011 | 0·138 ±0·05 | 0·09 ±0·015 | 0·14 ±0·025 | 0·102 ±0·055 | 0·34 ±0·07 | 0·225 ±0·04 |

Activities of all the phosphatases expressed in terms of μg. of phosphorus liberated per μg. of tyrosine.

Fibrosarcomas in both the strains have higher acid phosphatase and lower alkaline phosphatase activity than the mammary carcinomas. Thus the ratio of alkaline to acid phosphatase activity drops down as low as 0·17–0·18. Adenosine triphosphatase activity of fibrosarcomas in both the strains is significantly higher than that of the mammary carcinomas.

Table III shows the levels of ribo- and desoxyribonucleic acids of carcinomas of different tissue origins. Once again it is proved that nucleic acid levels of tumours do not vary with tissue origin. The same is the case of ribonuclease activity.

Table IV shows the activities of acid and alkaline phosphatases and adenosine triphosphatase activity of carcinomas of different tissue origin. It may be observed that liver tumours in Strong A and ICRC strains have high alkaline

TABLE III.—*Contents of ribo- and deoxyribonucleic acids and ribonuclease activity of first generation carcinomas of different tissue origin in different strains of mice*

| | Liver (Strong A) | Liver (I.C.R.C.) | Cervix (Strong A) | Lung (Swiss) | Ascites (Strong A) |
|---|---|---|---|---|---|
| RNA | 0·156 ±0·008 | 0·175 ±0·02 | 0·200 ±0·01 | 0·16 ±0·01 | 0·180 ±0·03 |
| DNA | 0·300 ±0·02 | 0·390 ±0·04 | 0·600 ±0·04 | 0·39 0·03 | 0·523 ±0·08 |
| RNA/DNA | 0·531 | 0·448 | 0·33 | 0·41 | 0·34 |
| RNAase | 0·09 ±0·02 | 0·108 ±0·04 | 0·158 ±0·06 | — | 0·100 ±0·027 |

Contents of nucleic acids expressed in μg. of nucleic acids per μg. of tyrosine.

RNAase activity expressed as μg. of nucleotides liberated per μg. of tyrosine.

TABLE IV.—*Activities of acid, alkaline phosphatase and adenosine triphosphatase of first generation carcinomas of different tissue origin in different strains of mice*

| | Liver (A) | Liver (I.C.R.C.) | Cervix (A) | Lung (Swiss) | Ascites (A) |
|---|---|---|---|---|---|
| Acid phosphatase | 0·384 ±0·02 | 0·460 ±0·045 | 0·283 ±0·012 | 0·168 ±0·08 | 0·320 ±0.05 |
| Alkaline phosphatase | 0·86 ±0·016 | 1·82 ±0·28 | 0·082 ±0·017 | 0·207 ±0·08 | 0·248 ±0·06 |
| Alkaline phosphatase/ acid phosphatase | 2·2 | 4·0 | 0·29 | 1·2 | 0·76 |
| ATPase | 0·120 ±0·05 | 0·07 ±0·017 | 0·156 ±0·014 | 0·125 ±0·009 | 0·092 ±0·02 |

Activities of all the phosphatases expressed in terms of μg. of phosphorus per μg. of tyrosine.

phosphatase activity and a high ratio of alkaline to acid phosphatase activity. The cervical tumour has higher acid phosphatase and lower alkaline phosphatase activity.

Lung and ascites tumour do not show significant differences in acid and alkaline phosphatase activity. Adenosine triphosphatase activity of these different carcinomas does not vary significantly.

### DISCUSSION

In the previous experiments we had observed that carcinoma (56th generation) had a lower RNA/DNA ratio and lower incorporation of labelled adenine into nucleic acids than the sarcoma (46th generation). But from the experiments reported above it is quite clear that all the tumour types studied display uniformity in nucleic acid levels as well as in RNA/DNA ratio. It seems that the earlier differences noted in two tumours were not due to their histological differences but due either to the extent of progression of these tumours or to their growth rate.

Differences in the activities of acid and alkaline phosphatases of mammary adenocarcinoma and fibrosarcomas are quite striking. Mammary adenocarcinomas possess very high alkaline phosphatase activity and a high ratio of alkaline to acid phosphatase activity. The normal mammary gland from which these tumours are derived also possesses remarkably high alkaline phosphatase activity (Holmes, 1956) ; the role of this enzyme in mammary gland is not yet elucidated. Folly (1949) has postulated that it may play an important role in lactose biosynthesis. It thus appears that even though the tissue has turned malignant it has still retained high enzyme activity. A similar instance where the constitutive enzymes are still maintained after malignant transformation is the slow growing Morris hepatoma (Weber, Banergee and Morris, 1961). This tumour possesses most of the glycolytic and gluconeogenic enzymes of the normal liver. Only fast growing hepatomas have shown deletion of constitutive enzymes (Weber, 1963). It is also interesting to note that mammary carcinomas of the breeders of all the three strains show a higher ratio of alkaline to acid phosphatase activity than for the corresponding virgin tumours. It was reported previously that alkaline phosphatase activity of mammary gland increases during pregnancy and lactation and then declines with involution (Folley and Greenbaum, 1947). It thus seems to be under the influence of mammotrophic and gonadotrophic hormones. It will therefore be interesting to study the behaviour of alkaline phosphatase in mammary gland and mammary tumour under various hormonal conditions.

The high acid phosphatase activity of fibrosarcomas in both the strains may also be noted. Characteristically both the tumours have a very low ratio of alkaline to acid phosphatase activity. Sarcomas also possess higher adenosine triphosphatase activity than mammary adenocarcinomas. It is known that muscle, the normal precursor of the tumour has very active ATPase. Higher adenosine triphosphatase activity in fibrosarcomas could also be due to their faster rate of growth and hence higher metabolic rate of the tumours. In the case of carcinomas of different tissues no definite correlation can be established with reference to phosphatases.

These observations point out the fact that carcinomas as a class cannot be tagged with biochemical characteristics. Neither nucleic acids nor nucleic acid ratio has shown any characteristic differences between carcinomas of different tissues and sarcomas. Significant differences are observed in the case of phosphatases. High alkaline phosphatase activity of mammary carcinomas seems to be the property of normal mammary tissue as well. It thus appears that useful information for biochemical characterisation of different tumours can be collected only when specific markers of the tissue of origin are explored. Extensive data on hepatomas has been collected in recent years. Weber (1962), Morris (1963) and Potter *et al.* (1960) have been able to correlate the enzymatic make-up of the hepatoma with its growth rate. Similar search for specific properties of tumours with reference to the parent tissue will help a great deal to have a clearer vision of the biochemical nature of the tumours.

### SUMMARY

Mammary carcinomas in different strains of mice, carcinomas of different tissue origins and sarcomas in two strains were studied for (i) nucleic acid levels,

(ii) acid ribonuclease activity, (iii) acid and alkaline phosphatases, (iv) adenosine triphosphatase activity. It was observed that nucleic acid content and acid ribonuclease activity of all the tumours studied did not show significant differences.

Mammary carcinomas had significantly higher alkaline phosphatase and lower acid phosphatase activity than fibrosarcomas. Other carcinomas of different tissue origin did not show any specific correlationship with phosphatase levels.

### REFERENCES

Ceriotti, G.—(1952) *J. biol. Chem.*, **198**, 297.
Divekar, M. V. and Bhide, S. V.—(1962) *Indian J. med. Sci.*, **16**, 324.
Folly, S. J.—(1949) *Biol. Rev.*, **24**, 316.
Folly, S. J. and Greenbaum, A. L.—(1947) *Biochem. J.*, **41**, 261.
Holmes, R. L.—(1956) *Nature, Lond.*, **178**, 311.
Krishnan, P. S.—(1955) *Meth. Enzym.*, **2**, 591.
Lowry, O. H., Rosenbourgh, N. J., Farr, A. L. and Randall, R. J.—(1951) *J. biol. Chem.*, **193**, 265.
Morris, H. P.—(1963) *Prog. exp. Tumor Res.*, **3**, 370.
Ogur, M. and Rosen, G.—(1950) *Archs Biochem.*, **25**, 262.
Potter, V. R., Pitot, H. C., Ono, T. and Morris, H. P.—(1960) *Cancer Res.*, **20**, 1255.
Ranadive, K. J., Kamat, K. A., Coutinho, T. C. and Khanolkar, V. R.—(1961) *Indian J. med. Res.*, **49**, 562.
Weber, G., Banergee, G. and Morris, H. P.—(1961) *Cancer Res.*, **21**, 933.
Weber, G.—(1962) *Adv. Cancer Res.*, **6**, 403.—(1963) *Adv. Enzyme Regulation*, **1**, 321.
Weil-Malherbe, H. and Green, R. H.—(1951) *Biochem. J.*, **49**, 286.